# The diagnostic value of third-generation nanopore sequencing in non-tuberculous mycobacterial infections

**DOI:** 10.3389/fcimb.2025.1557079

**Published:** 2025-04-01

**Authors:** Chun-Yan Zhao, Chang Song, Yan-Rong Lin, Ying-Xing Nong, Ai-Chun Huang, Shao-Yong Xi, Xiao-Ying Wei, Chun-Mei Zeng, Zhou-Hua Xie, Qing-Dong Zhu

**Affiliations:** ^1^ Department of Tuberculosis, The Fourth People’s Hospital of Nanning, Nanning, Guangxi, China; ^2^ Clinical Medical School, Guangxi Medical University, Nanning, Guangxi, China; ^3^ Department of Medical, The Fourth People’s Hospital of Nanning, Nanning, Guangxi, China; ^4^ Department of Clinical Laboratory, The Fourth People’s Hospital of Nanning, Nanning, Guangxi, China

**Keywords:** nanopore sequencing, NTMPD, diagnostic value, bacterial concentration, MTB infection

## Abstract

**Background:**

This study aimed to investigate the diagnostic value of nanopore sequencing technology in non-tuberculous mycobacterial pulmonary disease (NTMPD) and compare it with traditional culture methods.

**Methods:**

A retrospective analysis was conducted on 225 suspected NTMPD patients admitted to the Fourth People’s Hospital of Nanning City from January 2022 to July 2024. The sensitivity, specificity, positive predictive value (PPV), negative predictive value (NPV), kappa coefficient, and area under the receiver operating characteristic curve (AUC) of nanopore sequencing, culture, and combined diagnostic methods were compared to evaluate their diagnostic performance. In addition, patients were divided into different groups to investigate the detection of NTMPD by nanopore sequencing technology under different pathogen concentrations, in cases of concurrent Mycobacterium tuberculosis (MTB) infection, and among the elderly (aged > 60 years).

**Results:**

Among 139 NTMPD samples, nanopore sequencing detected positives in 113 cases, with a sensitivity of 81.3%, PPV of 99.1%, NPV of 76.6%, kappa coefficient of 0.759, and AUC of 0.901, demonstrating high specificity (98.8%) comparable to culture. The combined diagnostic approach significantly improved the sensitivity (90.6%), NPV (98.4%), kappa coefficient (0.862), and AUC (0.942) of NTMPD diagnosis. Nanopore sequencing showed superior diagnostic value in samples with various bacterial concentrations and in cases of concurrent MTB infection.

**Conclusion:**

Third-generation nanopore sequencing technology serves as a rapid and effective diagnostic tool, which may profoundly impact the current diagnosis of NTMPD.

## 1 Introduction

Non-tuberculous mycobacteria (NTM) constitute a group of mycobacteria other than Mycobacterium tuberculosis and Mycobacterium leprae (Jamal and Hammer, 2022). NTM is free-living and ubiquitous in the natural environment, inhabiting water systems, soil, and vegetation (Falkinham, 2013). While NTM was previously considered a secondary pathogen like Mycobacterium tuberculosis (MTB), NTMPD has garnered increasing attention over the past 30 years (Griffith and Aksamit, 2016). Several studies have shown that in 16% of geographical regions globally, there is a trend opposite to that of tuberculosis, particularly in developed countries where the incidence of NTMPD exceeds that of tuberculosis, including Japan, the United States, and Australia (Chin et al., 2020). The global incidence and prevalence of NTMPD are rising due to continuously improving monitoring and diagnostic techniques, broader population surveys, and the influence of human activities and environmental changes, necessitating urgent treatment and prevention efforts (Stout et al., 2016; Wassilew et al., 2016; Kumar and Loebinger, 2022). Recent reports indicate the possibility of person-to-person transmission (Bryant et al., 2016). Therefore, the control and management of NTMPD can become one of the significant global health challenges.

The early and accurate diagnosis of NTMPD is critical for disease treatment and epidemic control. Whereas NTMPD diseases are unresponsive to anti-tuberculosis drugs with treatment strategies varying with bacterial species, often requiring at least 18 months of therapy and 12 months of negative sputum smears (Gopalaswamy et al., 2020). Because of the morphological similarities between NTM and MTB, their differentiation through commonly used traditional acid-fast staining microscopy methods is challenging. Due to the NTM’s slow growth, traditional culture methods require a long time to yield reliable results. NTM is usually present at low concentrations in samples, resulting in a low culture positivity rate and complicating the diagnosis further. Additionally, many clinical symptoms of NTMPD diseases are like tuberculosis, exacerbating the diagnostic process. The challenge of diagnosing NTMPD is further complicated by the fact that individuals may carry these bacteria due to environmental exposure, requiring physicians to distinguish between NTM isolates as commensals or pathogens. This process is significantly more complex than tuberculosis (Gopalaswamy et al., 2020). Due to these characteristics, NTMPD is prevalent globally, leading to a series of misdiagnoses, confirmation difficulties, and treatment challenges, making therapeutic approaches for this disease difficult and complex (Ratnatunga et al., 2020).

Therefore, to improve the diagnostic accuracy and efficiency of NTMPD infections, it is critical to develop rapid and sensitive detection methods. The emergence of third-generation nanopore sequencing technology may revolutionize NTMPD diagnostics. Through real-time analysis of single molecules, nanopore sequencing technology is poised to achieve breakthroughs in speed and sensitivity levels that traditional culture methods cannot attain (Sun et al., 2020; Wang et al., 2021; Pugh, 2023). This technology not only accelerates the analysis and reduces the diagnostic duration but also improves NTMPD ‘s specific molecular marker identification through high-throughput data generation. Its unique sequencing approach can make it a powerful tool for the rapid diagnosis of NTMPD infections, improving the pathogen detection process, providing molecular diagnostic evidence for personalized treatment of patients, and ultimately achieving better treatment outcomes and quality of life.

Although several studies have explored the diagnostic value of nanopore sequencing in Mycobacterium tuberculosis, to our knowledge, there have been no reports on the diagnostic potential of third-generation nanopore sequencing technology for NTMPD. Therefore, by integrating the clinical characteristics of patients, different bacterial concentrations, co - infection with Mycobacterium tuberculosis (MTB), and the results from the elderly patient population, we aim to evaluate the effectiveness and accuracy of nanopore sequencing as a diagnostic tool for NTMPD.

## 2 Material and methods

### 2.1 Subject recruitment

A retrospective cohort analysis was conducted on 225 suspected cases of NTMPD admitted to the Fourth People’s Hospital of Nanning City from January 2022 to July 2024. The inclusion criteria were as follows: (1) Patients with typical lung lesions indicative of NTM infection detected using radiological examination, including bronchiectasis, nodules or nodular lesions, fibro cavitary lesions, inflammatory infiltrates and nodules, miliary lesions, or calcified foci; (2) patients with persistent symptoms like chronic cough, sputum production, dyspnea, chest pain, weight loss, fatigue, and fever, with ineffective anti-tuberculosis and conventional antibacterial treatment; (3) patients with initial acid-fast staining positive results but ineffective anti-tuberculosis treatment. The exclusion criteria were as follows: (1) Patients who did not undergo third-generation nanopore sequencing and culture; (2) patients with incomplete clinical data; (3) patients who only underwent either third-generation nanopore sequencing or culture; (4) patients or their legal guardians who did not sign informed consent; (5) patients with unclear final clinical diagnosis.

The final diagnosis of NTMPD is determined in accordance with the 2020 American Thoracic Society (ATS) “Guidelines for the Treatment of NTM Disease” (Daley et al., 2020), the 2017 British Thoracic Society (BTS) “Guidelines for the Management of NTMPD “ (Haworth et al., 2017) and the Chinese Medical Association’s Diagnosis and Treatment Guidelines for Non-tuberculous Mycobacterial Diseases (2020 version) (Chinese Society for Tuberculosis, 2020). Patients with respiratory symptoms and/or systemic symptoms, who have cavitary shadows, multifocal bronchiectasis, and multiple small nodular lesions detected by chest imaging, and in whom other pulmonary diseases have been excluded, can be diagnosed with NTMPD if any of the following criteria are met under the premise that the specimen is free from exogenous contamination: (1) Two separate sputum specimens are positive for NTM culture and identified as the same pathogen, and/or NTM molecular biological tests are positive for the same pathogen. (2) NTM culture and/or molecular biological tests of bronchoalveolar lavage fluid (BALF) or bronchial washing fluid are positive. (3) Histopathological features of mycobacterial disease (granulomatous inflammation or acid-fast staining positivity) are found in lung tissue biopsy obtained via bronchoscopy or other means, and NTM culture and/or molecular biological tests are positive. (4) Histopathological features of mycobacterial disease (granulomatous inflammation or acid-fast staining positivity) are found in lung tissue biopsy obtained via bronchoscopy or other means, and NTM culture and/or molecular biological tests are positive in one or more sputum specimens, BALF, or bronchial washing fluid. In this study, a positive result was defined as a final diagnosis of NTMPD, whereas a negative result was defined as the absence of a diagnosis of NTMPD.

The final clinical diagnosis of each pulmonary tuberculosis patient was determined according to the “WS 288-2017 Pulmonary Tuberculosis Diagnostic Criteria” issued by the Chinese Center for Disease Control and Prevention. This study was approved by the Human Research Ethics Committee of the Fourth People’s Hospital of Nanning City (Ethics Approval No. [2023]24). All participants or their legal guardians provided informed consent. Moreover, all researchers ensured that the planning, implementation, and reporting of this study complied with the revised Helsinki Declaration of 2013.

### 2.2 Sample collection

Morning sputum samples were collected. Based on the location of respiratory tract lesions, BALF samples containing cells and secretions from the alveoli, which could be potential hosts for mycobacteria, were obtained using endobronchial ultrasound technology. We strictly adhere to standardized procedures for sample collection and testing, ensuring that every step is carried out with precision and accuracy. This approach effectively minimizes deviations caused by differences in sample handling, thereby guaranteeing the reliability and accuracy of the test results.

### 2.3 Acid-fast Bacillus culture

An equal volume of 4% NaOH solution was added to approximately 5 mL of the sample, vortexed for 20 s, and allowed to rest at room temperature for 15 min. A phosphate buffer solution with a pH of 7.2 was added, and the mixture was centrifuged and precipitated. The total processing time should not exceed 20 min. Many samples should be processed in batches. Modified Loewenstein culture medium (Beisen Technology Co., Ltd., Zhuhai, China; specification: 50 tubes/box) was used. The culture medium was aseptically inoculated with 0.1 mL of the pretreated sample, and the inoculated slant was shaken to evenly spread the liquid. The bottle cap was secured and incubated flat at 35 °C for 24 h, and then the slant was positioned upright and incubated at 35 °C. The samples were observed three and seven days after inoculation, followed by weekly observations. Positive smear results were reported at any time. A positive result reported within seven days indicates rapid-growing bacteria, while one reported after seven days indicates slow-growing bacteria. Negative results were reported after eight weeks, with possible extension if necessary. Clinical samples should be handled in a biosafety level 2 cabinet as a principle, and contamination should be rigorously avoided. When observing the growth of mycobacteria, if other pathogens are detected, it is reported as contaminated and retested. The contamination rate should be controlled below 2%; if it exceeds 2%, it indicates contamination of the culture medium or improper sample handling.

### 2.4 Nanopore sequencing

#### 2.4.1 Sequencing protocol expansion

Added explicit library preparation steps (DNA repair, adapter ligation, purification) with commercial kit references (ONT SQK-LSK114, NEB enzymes) to ensure reproducibility. Specified flow cell type (R10.4.1) and voltage parameters, as these critically impact read accuracy and throughput.

#### 2.4.2 Software versioning and parameters

Detailed basecalling tools (Guppy v6.4.6) and quality filters (Q-score ≥7) to align with ONT’s latest recommendations. Included post-processing tools (Porechop, NanoFilt, Kraken2) and their parameters to clarify how raw data were refined before analysis.

#### 2.4.3 Database and thresholds for pathogen detection

Defined the microbial reference database (NCBI RefSeq) and statistical criteria for species identification (50% identity, 10× coverage) to address potential false-positive concerns.

In our research, we took the following quality control steps: First, to minimize errors, we used the high - precision model of the latest Guppy base - calling algorithm provided by Oxford Nanopore Technologies to improve the accuracy of base calling. Meanwhile, we performed read filtering by removing low - quality reads with a length of less than 200 nucleotides or more than 1000 nucleotides and a quality score (Q value) of less than 7. In addition, we used the minimap2 tool to align the reads with the reference genome. This tool is optimized for long reads and can effectively identify and filter out reads with alignment errors or structural variations that may lead to errors in variant detection. Second, we combined the read length and quality score thresholds to further filter low - quality reads. We excluded reads shorter than 200 nucleotides or longer than 2000 nucleotides and those with an average Phred quality score (Q value) below 7 to ensure that only high - quality reads were used for downstream analysis. Finally, we calculated the coverage depth of the samples using the Samtools depth function. The average coverage depth was between 90 - fold and 1000 - fold, with a median of 325 - fold. We also visually inspected the distribution of the whole - genome coverage to ensure the uniformity of coverage.

### 2.5 Data processing and analysis

Statistical analysis was performed using SPSS 23.0 software. The indicators for evaluating diagnostic performance included sensitivity, specificity, PPV, NPV, kappa coefficient, and AUC. To compare the diagnostic performance of nanopore sequencing and solid culture of mycobacteria, McNemar’s chi-square test was used to analyze paired data. P < 0.05 was considered statistically significant.

## 3 Results

### 3.1 Clinical characteristics of participants

**Figure 1:**
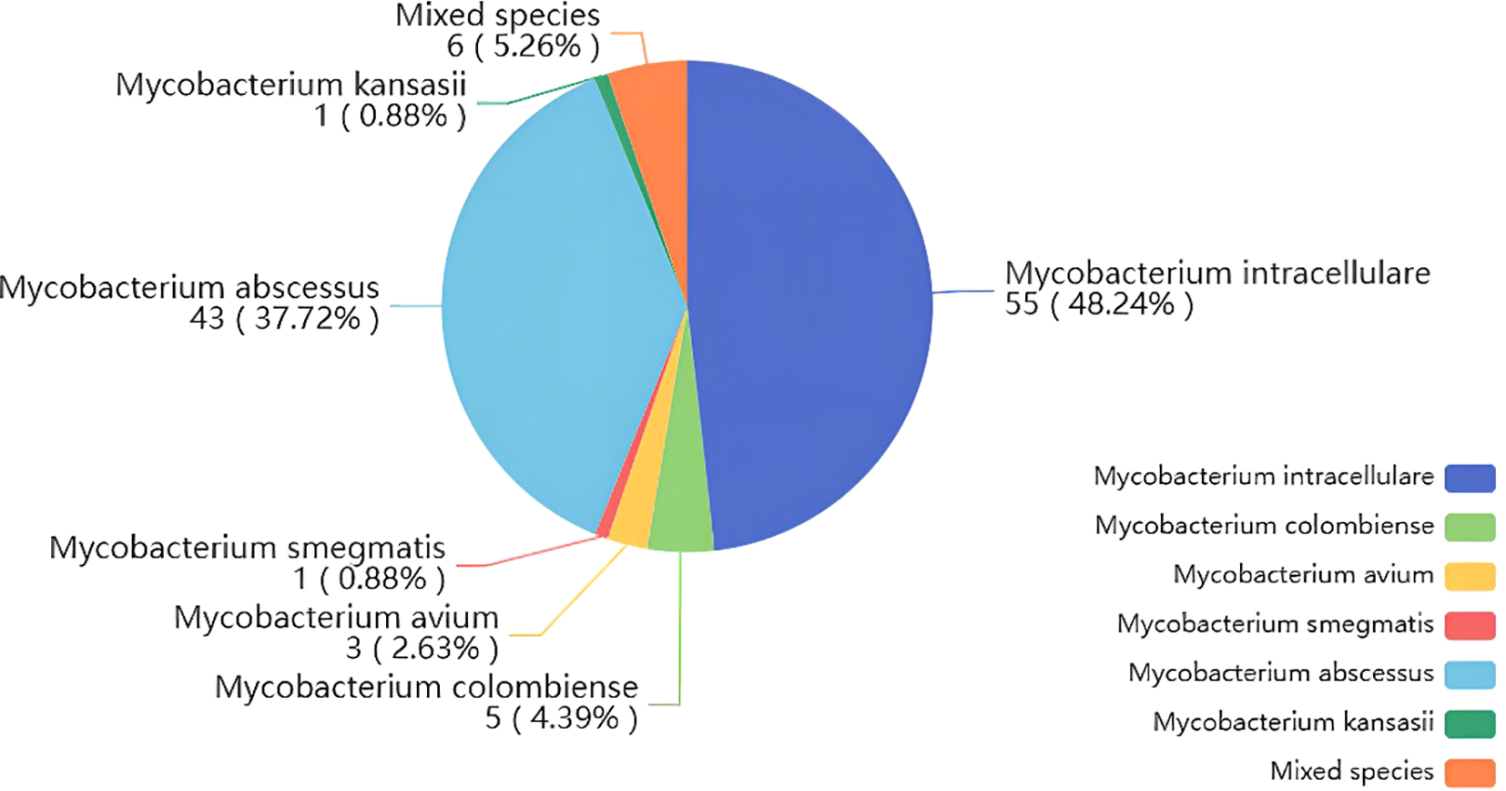
Pie chart of the composition ratio of NTM species in the cases diagnosed by nanopore sequencing diagnosis.

This study included 225 individuals, among whom 139 were diagnosed with NTMPD. The diagnosed patients included 48 males (34.53%) and 91 females (65.47%). This finding is consistent with previous studies, indicating that elderly females were more susceptible to the disease (Ku et al., 2020). Among the 225 included samples, 176 were from BALF and 49 were from sputum. Among the 139 NTMPD-positive samples, 50 were co-infected with Mycobacterium tuberculosis, and 81 had bronchiectasis. Specific information on other complications is presented in **Table 1**. In the NTMPD group, the most common species was Mycobacterium intracellular (48.25%), followed by Mycobacterium abscesses (37.72%), and there were also 6 cases of mixed nontuberculous mycobacterial infections (5.26%) (**Figure 1**).

**Table 1:** Clinical characteristics of the included patients.

	NTMPD(n=139)	Non-NTMPD(n=86)
Gender		
Male	48	55
Female	91	31
Age (years) (mean ± SD)	56.95 ± 14.81	59.82 ± 13.23
Complications		
Tuberculosis	50	66
Bronchiectasis	81	67
Chronic obstructive pulmonary disease	9	18
Pleural disorders	11	19
Pneumonia or upper respiratory tract inflammation	25	33
Respiratory failure	11	13
Pleural effusion	9	3
Pulmonary arterial diseases (hypertension/fistula)	3	3
Lung cancer	1	2
Sample type		
BALF	106	70
Sputum	33	16

### 3.2 Efficacy of nanopore sequencing, culture, and combined diagnosis

Among the 139 NTMPD samples, 113 were detected as positive by nanopore sequencing, whereas 93 were detected as positive by culture (**Table 2**). Nanopore sequencing demonstrated superior sensitivity (81.3%), PPV (99.1%), NPV (76.6%), and Kappa coefficient (0.759) while maintaining a similarly high specificity (98.8%) as culture. Furthermore, combining nanopore sequencing with culture significantly improved the indicators.

**Table 2:** Diagnostic efficacy of nanopore sequencing, culture and combined diagnosis.

	Non-NTMPD	NTMPD	Sensitivity	Specificity	PPV	NPV	Kappa	P value
Nanopore sequencing assay								
Negative	85	26	81.30%	98.80%	99.10%	76.60%	0.759	<0.01
Positive	1	113						
Culture								
Negative	85	46	66.90%	98.80%	98.90%	64.90%	0.598	<0.01
Positive	1	93						
Nanopore sequencing assay & Culture								
Negative	84	13	90.60%	97.70%	98.40%	86.60%	0.862	<0.01
Positive	2	126						

**Figure 2:**
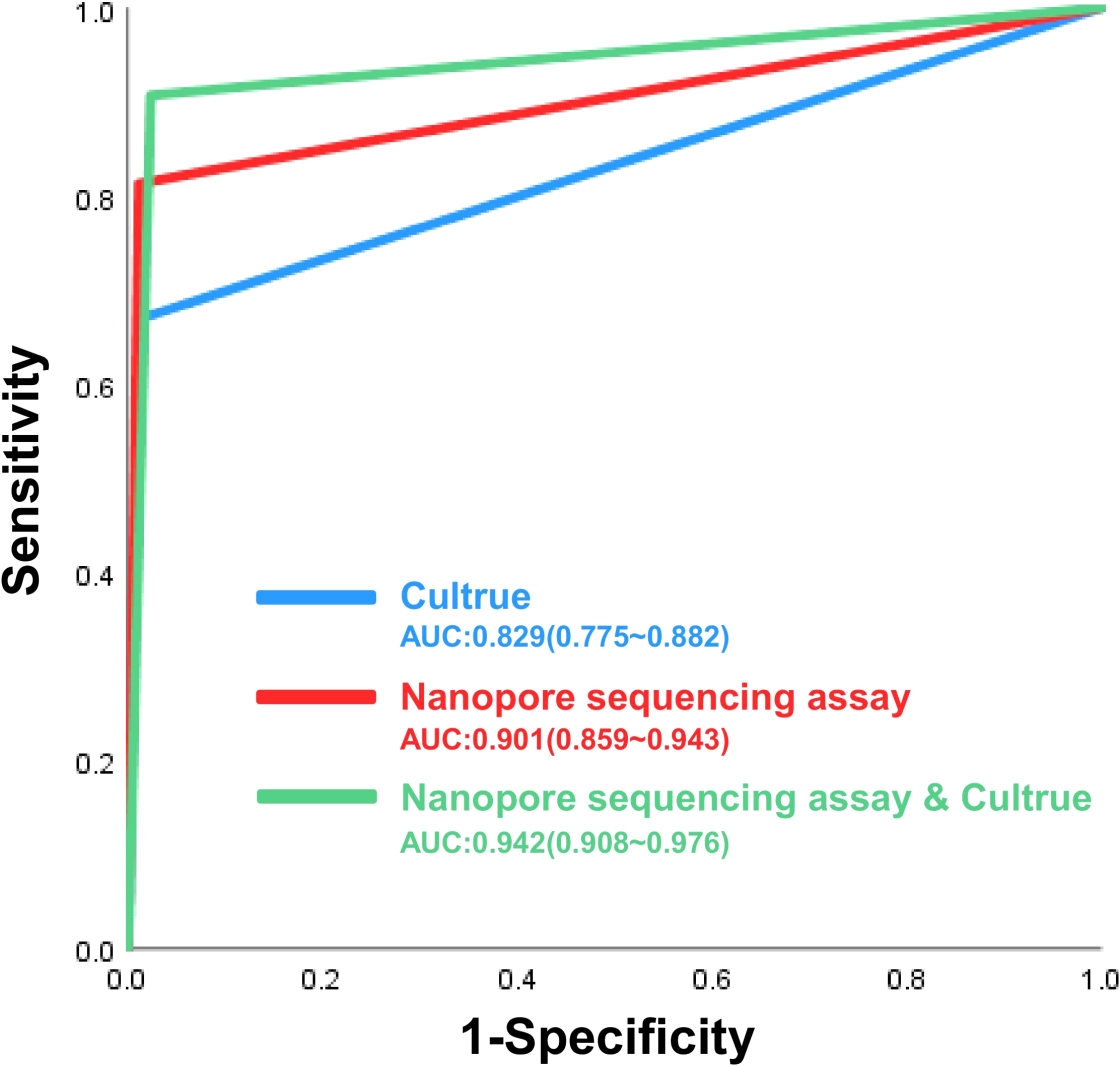
The receiver operating characteristic (ROC) curves of nanopore sequencing, culture and Nanopore sequencing in the diagnosis of NTMPD.

The ROC curves for the various diagnostic methods are presented in **Figure 2**. The AUC for Nanopore sequencing assay and culture was 0.942 (95% CI: 0.908–0.976), which was higher than that of culture alone (0.829 [95% CI: 0.775–0.882]) and standalone nanopore sequencing (0.901 [95% CI: 0.859–0.943]). All P-values were less than 0.05, indicating statistical significance.

### 3.3 Venn diagram of nanopore sequencing and culture-obtained NTM positive result

**Figure 3:**
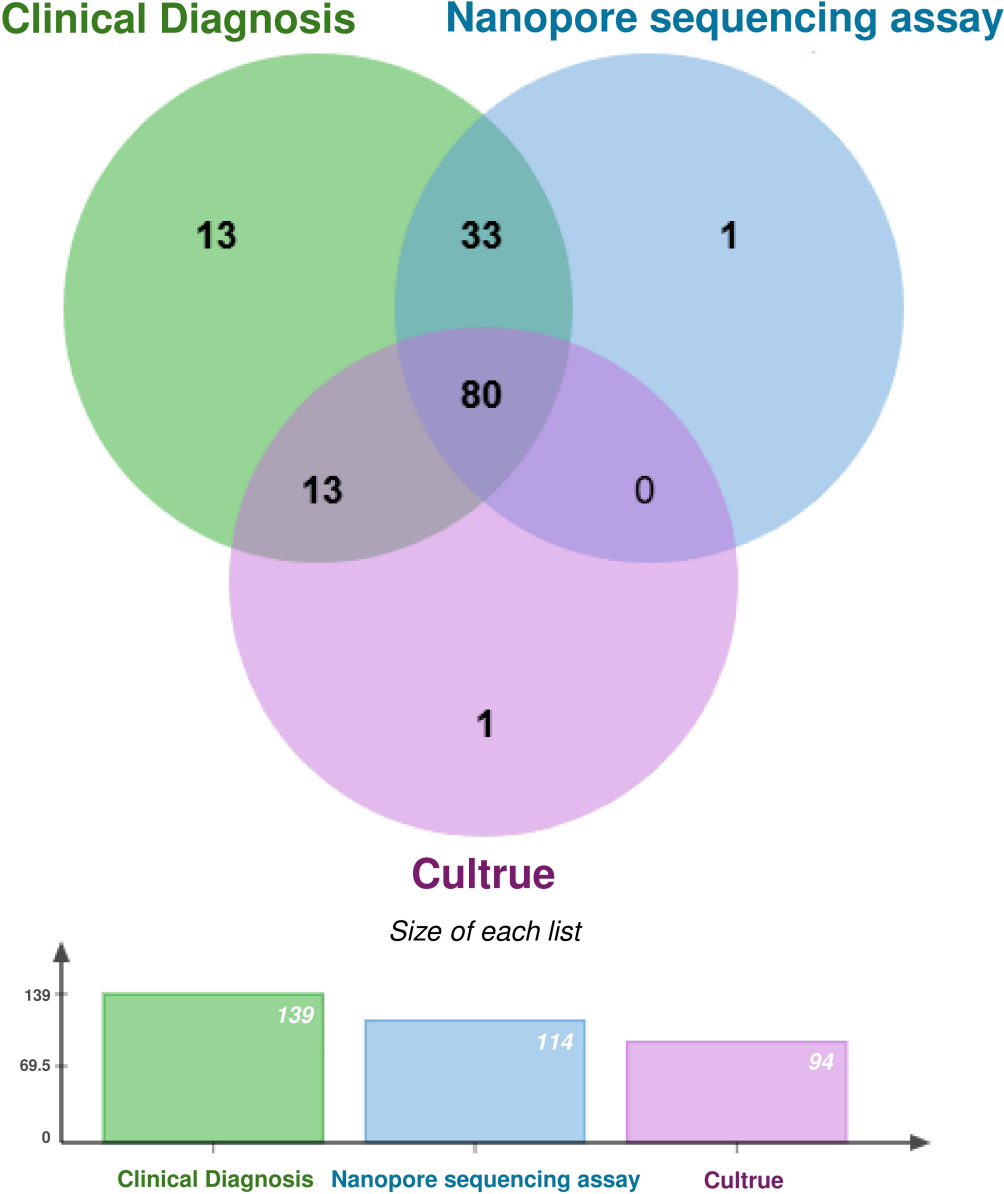
Venn diagram of NTM positive results obtained by nanopore sequencing and culture.

All 139 specimens from NTMPD individuals were subjected to nanopore sequencing and culture, yielding 113 positive results (81.29%) and 93 positive results (66.91%), respectively (**Figure 3**). It is noteworthy that nanopore sequencing successfully identified 33 positive cases missed by culture. However, 13 positive cases detected by culture were overlooked by nanopore sequencing.

### 3.4 Detection of NTM based on the number of pathogenic bacterial sequences

**Figure 4:**
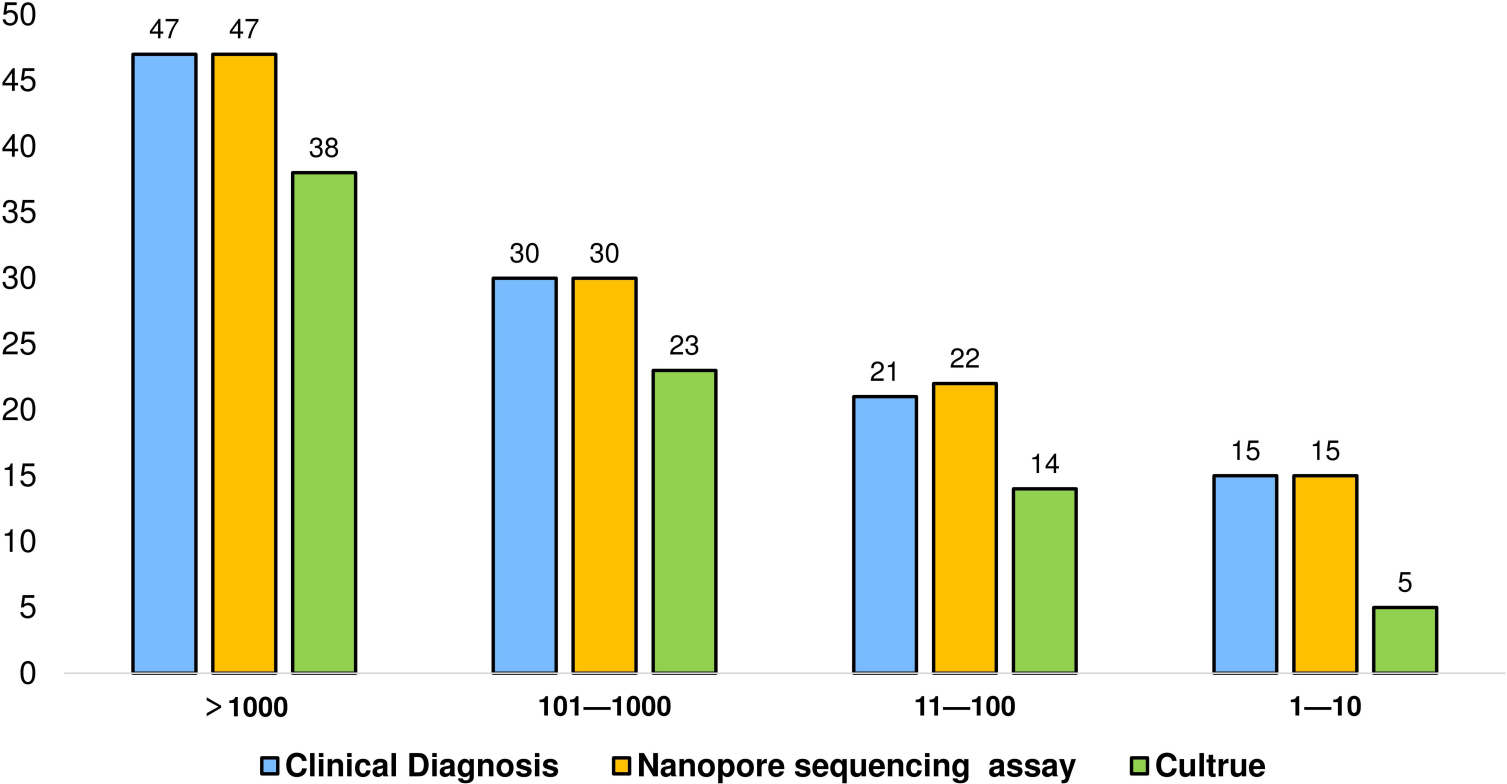
Positive detection of different pathogen sequence numbers by nanopore sequencing, culture and γ interferon test (Samples with untyped, mixed infection and unknown final sequence number were not included).

We classified the pathogenic bacterial sequences into four groups using third-generation nanopore sequencing technology: >1000, 101–1000, 11–100, and 1–10. The positive detection rates of nanopore sequencing assays and culture were compared in different groups. The results revealed that in the groups with >1000 and 100–1000 pathogenic bacterial sequences, culture failed to detect 9 and 7 positive results, respectively. In the group with 10–100 sequences, nanopore sequencing detected 22 positive results (including one false positive), while in the group with 1–10 sequences, culture showed lower positive detection rates, detecting only 5 positive cases (**Figure 4**).

### 3.5 Impact of Mycobacterium tuberculosis on NTM detection

**Figure 5:**
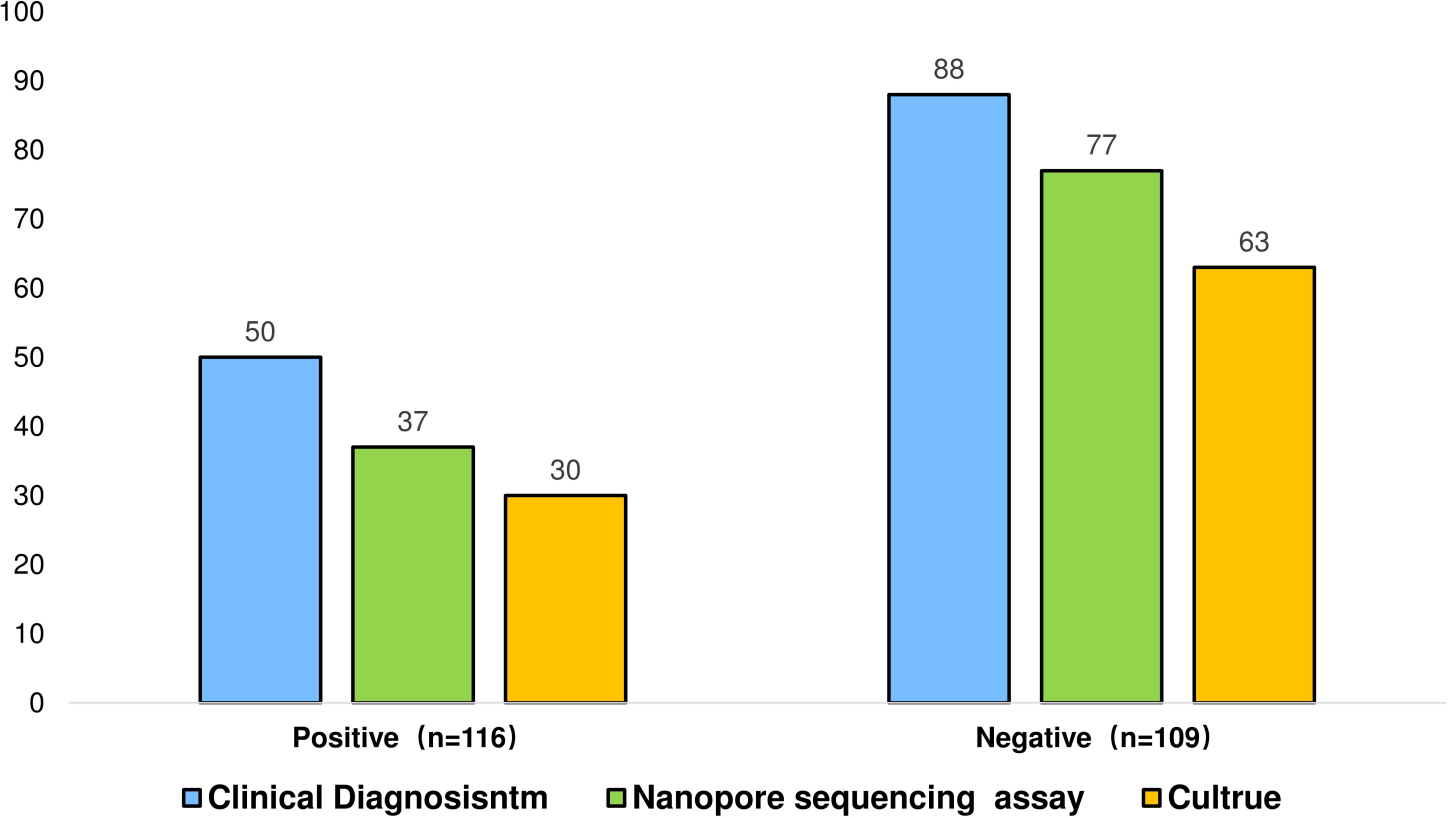
Positive detection of MTB infection by nanopore sequencing, culture method and gamma interferon assay.

To investigate the interference effect of Mycobacterium tuberculosis (MTB) sequences on NTM detection, samples were divided into two groups based on MTB infection status: MTB-positive (n=116) and negative (n=109). NTM detection rates between the two groups were then compared under different conditions. The results indicate that when considering the influence of MTB infection, nanopore sequencing technology exhibited a higher detection rate in the MTB-positive group (74%) than in the detection rate of culture (60%), demonstrating its superiority (**Figure 5**). In the case of simple NTM infection, the detection rate of NTM by nanopore sequencing is 87.5%, while that by culture is 71.59%.

### 3.6 Detection efficiency of nanopore sequencing for NTM in different samples and the elderly

Among the 225 patients included in the study, 49 cases were sputum samples, and 176 cases were BALF samples. In the comparison of the detection efficacy of these two sample types, nanopore sequencing showed higher sensitivity (83%), NPV (NPV, 79.3%), and Kappa coefficient (0.784) in BALF samples than in sputum samples (sensitivity 75.8%, NPV 66.7%, Kappa coefficient 0.671). However, in terms of specificity and positive predictive value (PPV), sputum samples performed better, with both specificity and PPV reaching 100%, while the specificity of BALF samples was 98.6% and the PPV was 98.9%. This difference was mainly due to a false - positive result in the BALF samples (**Supplementary Tables 1**, **2**). When further analyzing the elderly patient group, nanopore sequencing was superior to the overall level in all indicators of NTMPD detection. Specifically, the sensitivity was 84.5%, the specificity was 100%, the PPV was 100%, the NPV was 78.4%, and the Kappa coefficient was 0.797 (**Supplementary Table 3**). This indicates that nanopore sequencing has higher diagnostic efficacy in elderly patients.

## 4 Discussion

Recently, the incidence and prevalence of pulmonary NTM-related cases have increased globally (Matsuyama et al., 2023). Therefore, it is imperative to improve the understanding, prevention, diagnosis, and treatment of pulmonary NTM disease, especially in tropical and economically underdeveloped countries where NTM is more prevalent (Ratnatunga et al., 2020). Due to its ease of use and versatility, nanopore sequencing is increasingly used in epidemiological research (Lewandowski et al., 2019; Schmidt et al., 2020; Sheka et al., 2021; Tourancheau et al., 2021). Several studies have evaluated the significance of third-generation nanopore sequencing in tuberculosis diagnosis (Yu et al., 2022; Liu et al., 2023; Yang et al., 2023); however, its potential in the diagnosis of NTMPD has not yet been proven.

In this study, among 139 cases of NTMPD, the most common co - existing pulmonary disease was bronchiectasis, followed by Mycobacterium tuberculosis infection, pneumonia, and upper respiratory tract inflammation. There is a close bidirectional relationship between NTM infection and bronchiectasis. The incidence of NTM infection is on the rise globally, especially in Western countries. The common types of NTMPD include the classic cavitary form and the atypical bronchiectasis form, with the latter more likely to occur in elderly women without a history of pulmonary disease (Jamal and Hammer, 2022). Patients with bronchiectasis are more susceptible to NTM infection due to damaged airway structures and impaired mucus clearance, and NTM infection, in turn, can further exacerbate the condition of bronchiectasis (Youssefnia et al., 2022), creating a vicious cycle. Moreover, even after NTM infection is cured, bronchiectasis still requires long-term management (McShane, 2023). Therefore, identifying the presence of NTM infection is crucial for differentiating between simple bronchiectasis and NTM infection-associated bronchiectasis. Detecting the species and drug resistance of NTM helps to formulate targeted treatment plans, avoid unnecessary use of broad-spectrum antibiotics, and thereby reduce the risk of drug resistance and improve treatment efficacy.

In this study, nanopore sequencing and culture were used to identify 139 NTMPD samples. The results revealed that nanopore sequencing detected 114 positive cases, with only 1 false positive, while culture detected 94 positive cases, with 1 false positive. Nanopore sequencing demonstrated higher sensitivity (81.30%), PPV (99.10%), NPV (76.60%), and Kappa coefficient (0.759) compared to culture while maintaining similarly high specificity (98.80%). The combined diagnosis achieved a sensitivity of 90.60%, with a NPV of 86.6%, and a kappa coefficient of 0.862. Furthermore, different diagnostic methods were compared by plotting the ROC curves. The combined application of nanopore sequencing and culture method was 0.942 (95% CI: 0.908 - 0.976), which was significantly higher than that of the culture method used alone (AUC = 0.829, 95% CI 0.775 - 0.882) and the nanopore sequencing method used alone (AUC = 0.901, 95% CI: 0.859 - 0.943). The combination of nanopore sequencing and traditional culture methods offers significant advantages in clinical diagnostics, especially in detecting low-abundance pathogens. Nanopore sequencing, with its rapidity, high sensitivity, and long-read capabilities, can quickly identify potential pathogens, compensating for the time-consuming nature of traditional culture. Meanwhile, culture methods provide precise quantification and validation through live bacterial isolation, ensuring the reliability of diagnostic results. This integration not only enhances diagnostic accuracy and efficiency but also provides robust support for detecting complex and polymicrobial infections. It demonstrates unique value in rapid diagnosis, dynamic monitoring, and evaluating treatment efficacy, offering an important direction for the future development of pathogen detection technologies.

Both culture methods and nanopore sequencing have encountered false positives and false negatives. The possible reasons for these results include false positives mainly stem from non-specific amplification or contamination, limitations of bioinformatics analysis, and misidentification due to the high genetic homology between NTM and Mycobacterium tuberculosis; false negatives may be caused by insufficient detection capability for low-abundance samples, inadequate sequencing depth, and nucleic acid degradation due to improper sample handling.

In 2019, Wei Gu et al. proposed that in clinical metagenomic next-generation sequencing (NGS), counting sequencing reads can provide quantitative or semi-quantitative data on the concentration of organisms in samples, especially for polymicrobial samples or disease processes involving multiple pathogens (Gu et al., 2019). As early as 2014, a case report also mentioned that NGS can comprehensively describe the bacteria causing patient infections. Moreover, its results are consistent with traditional 16S rRNA gene sequencing and can accurately determine the sources of most deep sequencing reads (Salipante et al., 2014). In this study, different pathogen sequences were classified using third-generation nanopore sequencing technology. The results revealed that in the high-concentration pathogen sequence group, culture failed to detect certain pathogens, while nanopore sequencing technology exhibited greater accuracy. Nanopore sequencing demonstrated a higher positive detection rate in the low-concentration sequence group. This study suggests that third-generation nanopore sequencing technology may be a more sensitive tool for identifying and classifying pathogens, especially in cases where traditional diagnostic methods fail to detect low-concentration pathogens.

Conventional diagnostic methods may not accurately distinguish infections caused by different pathogens, where the symptoms and signs of different pathogens may overlap, complicating the diagnosis (Pan et al., 2019). Recurrence is usually assumed when sputum smear/culture results are positive in patients with a history of tuberculosis; however, studies have revealed that a considerable proportion of infections are caused by non-tuberculous mycobacteria (Li et al., 2022). Patients might miss the optimal treatment window while awaiting further testing after culture. Therefore, to investigate the sequence noise interference effect of Mycobacterium tuberculosis sequences in the detection of non-tuberculous mycobacteria, the samples were divided into MTB-positive (n=116) and negative groups (n=109) according to MTB infection and compared the NTM detection rates under different conditions. In this study, only 30 positive cases were detected through culture among NTMPD patients with concurrent Mycobacterium tuberculosis infection, significantly lower than the NTM detection rate of nanopore sequencing technology (87.5%).

The nanopore sequencing had a detection rate 1.22 times higher than traditional culture methods in single-infection groups and 1.23 times higher in mixed-infection groups, with p-values less than 0.05, indicating a more significant advantage in mixed infections. This advantage stems from the limitations of traditional methods. For example, acid-fast staining cannot distinguish between MTB and NTM, while culture methods are time-consuming and have low positive rates. In cases of co-infection with TB and NTM, the rapid growth of MTB may inhibit the growth of NTM, leading to an underestimation of NTM culture positivity (Zaber et al., 2024). Therefore, nanopore sequencing has a greater advantage in detecting NTM, especially in the presence of MTB infection. The development of mycobacteriology relies on the combination of traditional methods and modern molecular techniques, with a focus on shortening diagnostic time and providing more accurate species identification and drug susceptibility results (O’Connor et al., 2015). As an emerging technology, nanopore sequencing has brought breakthroughs to the diagnosis of mycobacteria and is expected to play an important role in the future.

Nanopore sequencing holds great potential in clinical diagnostics but still faces challenges such as high error rates, low throughput, high costs, and complex bioinformatics analysis, especially in resource-limited settings. To address these issues, the following approaches can be considered: First, improve sequencing accuracy by optimizing nanopore design and signal processing algorithms. Second, reduce costs and simplify operational procedures by developing more affordable reagents and equipment. Third, enhance bioinformatics analysis capabilities through multidisciplinary collaboration. Fourth, prioritize the application of nanopore sequencing in high-demand scenarios, such as rapid pathogen detection and antimicrobial resistance analysis, where its advantages of long-read lengths and quick results can be fully utilized.

This study still had certain limitations. First, study has a relatively small sample size, which may have introduced statistical bias. Second, some NTMPD-positive results in the study were not accurately identified, which could be related to patients’ atypical clinical symptoms. Further research is required to determine whether nanopore sequencing can improve the prognosis of NTMPD patients.

## 5 Conclusion

Third-generation nanopore sequencing excels in diagnosing NTMPD with high accuracy and sensitivity. Combining it with culture improves its accuracy, especially in detecting low pathogen concentrations. It also detects NTM more accurately in patients co-infected with MTB, indicating a more precise identification and treatment. Overall, it is a promising diagnostic tool for NTMPD with the potential to improve medical diagnostics and patient outcomes.

## Data Availability

The original contributions presented in the study are included in the article/**Supplementary material**. Further inquiries can be directed at the corresponding authors. The data presented in the study are deposited in the SRA repository, accession number PRJNA1228325.
