# Supplementary material for: The diagnostic value of third-generation nanopore sequencing in non-tuberculous mycobacterial infections
Source: Front Cell Infect Microbiol. 2025 Apr 1;15:1557079. doi: 10.3389/fcimb.2025.1557079 (PMC11996914; doi:10.3389/fcimb.2025.1557079)
Supplement: Supplementary file 1 [file Table1.docx]

Supplementary Table 1. Diagnostic efficacy of nanopore sequencing for NTM in sputum samples

|  | Non-NTMPD | NTMPD | Sensitivity | Specificity | PPV | NPV | kappa | P value | AUC |
| --- | --- | --- | --- | --- | --- | --- | --- | --- | --- |
| **Nanopore sequencing assay** |  |  |  |  |  |  |  |  |  |
| Negative | 16 | 8 | 75.80% | 100.00% | 100.00% | 66.70% | 0.671 | ＜0.01 | 0.879（0.783~0.974） |
| Positive | 0 | 25 |  |  |  |  |  |  |  |
| **Cultrue** |  |  |  |  |  |  |  |  |  |
| Negative | 16 | 12 | 63.60% | 100.00% | 100.00% | 57.10% | 0.533 | ＜0.01 | 0.818（0.703~0.933） |
| Positive | 0 | 21 |  |  |  |  |  |  |  |
| **Nanopore sequencing assay & Cultrue** |  |  |  |  |  |  |  |  |  |
| Negative | 16 | 2 | 93.90% | 100.00% | 100.00% | 88.90% | 0.91 | ＜0.01 | 0.970（0.921~1.000） |
| Positive | 0 | 31 |  |  |  |  |  |  |  |

Supplementary Table 2. Diagnostic efficacy of nanopore sequencing for NTM in BALF samples

|  | Non-NTMPD | NTMPD | Sensitivity | Specificity | PPV | NPV | kappa | P value | AUC |
| --- | --- | --- | --- | --- | --- | --- | --- | --- | --- |
| **Nanopore sequencing assay** |  |  |  |  |  |  |  |  |  |
| Negative | 69 | 18 | 83.00% | 98.60% | 98.90% | 79.30% | 0.784 | ＜0.01 | 0.908（0.861~0.954） |
| Positive | 1 | 88 |  |  |  |  |  |  |  |
| **Cultrue** |  |  |  |  |  |  |  |  |  |
| Negative | 69 | 34 | 67.90% | 98.60% | 98.60% | 67.00% | 0.616 | ＜0.01 | 0.832（0.772~0.893） |
| Positive | 1 | 72 |  |  |  |  |  |  |  |
| **Nanopore sequencing assay & Cultrue** |  |  |  |  |  |  |  |  |  |
| Negative | 68 | 11 | 89.60% | 97.10% | 97.90% | 86.10% | 0.849 | ＜0.01 | 0.934（0.893~0.975） |
| Positive | 2 | 95 |  |  |  |  |  |  |  |

Supplementary Table 3. Diagnostic efficacy of nanopore sequencing for NTM in the elderly patient population (> 60 years old)

|  | Non-NTMPD | NTMPD | Sensitivity | Specificity | PPV | NPV | kappa | P value | AUC |
| --- | --- | --- | --- | --- | --- | --- | --- | --- | --- |
| **Nanopore sequencing assay** |  |  |  |  |  |  |  |  |  |
| Negative | 40 | 11 | 84.50% | 100.00% | 100.00% | 78.40% | 0.797 | ＜0.01 | 0.923（0.871~0.974） |
| Positive | 0 | 60 |  |  |  |  |  |  |  |
| **Cultrue** |  |  |  |  |  |  |  |  |  |
| Negative | 39 | 25 | 64.80% | 97.50% | 97.90% | 60.90% | 0.551 | ＜0.01 | 0.811（0.732~0.891） |
| Positive | 1 | 46 |  |  |  |  |  |  |  |
| **Nanopore sequencing assay & Cultrue** |  |  |  |  |  |  |  |  |  |
| Negative | 39 | 6 | 91.50% | 97.50% | 98.50% | 86.70% | 0.867 | ＜0.01 | 0.945（0.898~0.993） |
| Positive | 1 | 65 |  |  |  |  |  |  |  |
